# Reactive Oxygen Species, a Potential Therapeutic Target for Vascular Dementia

**DOI:** 10.3390/biom15010006

**Published:** 2024-12-25

**Authors:** Amanda Louise White, Grant M. Talkington, Blake Ouvrier, Saifudeen Ismael, Rebecca J. Solch-Ottaiano, Gregory Bix

**Affiliations:** 1 Clinical Neuroscience Research Center, Department of Neurosurgery, Tulane University School of Medicine, New Orleans, LA 70112, USA; 2 Tulane Brain Institute, Tulane University, New Orleans, LA 70112, USA; 3 Department of Microbiology and Immunology, Tulane University School of Medicine, New Orleans, LA 70112, USA; 4 Department of Neurology, Tulane University School of Medicine, New Orleans, LA 70112, USA; 5 School of Public Health and Tropical Medicine, Tulane University, New Orleans, LA 70122, USA

**Keywords:** vascular dementia, Alzheimer’s disease, antioxidants, reactive oxygen species, therapeutic

## Abstract

Vascular dementia (VaD) is a progressive neurodegenerative condition prevalent among elderly adults marked by cognitive decline resulting from injured and/or improperly functioning cerebrovasculature with resultant disruptions in cerebral blood flow. Currently, VaD has no specific therapeutics and the exact pathobiology is still being investigated. VaD has been shown to develop when reactive oxygen species (ROS) form from damaged targets at different levels of organization—mitochondria, endothelial cells, or cerebrovasculature. In this review, we highlight how specific ROS molecules may be important in the development of VaD and how they can be targeted as a potential therapeutic for VaD.

## 1. Introduction

Vascular dementia (VaD) is a condition marked by a progressive decline in cognitive function as a result of chronic interruptions in blood flow within the cerebrovasculature [1]. Despite the widespread prevalence and downstream consequences of this debilitating illness, no Food and Drug Administration (FDA)-approved treatments targeting the underlying etiology of VaD currently exist. This may be due to the various biological symptoms of VaD, as the exact pathobiology and root cause have yet to be defined [2]; however, many studies have implicated oxidative stress as a major player [3,4].

Oxidative stress is caused by the oversaturation of reactive oxygen species (ROS), which through inappropriate interactions and alterations can result in damage at the molecular and cellular levels [5]. ROS is a term used to refer to unstable molecules that contain oxygen and readily react with other molecules [6]. Low levels of ROS are produced as a natural byproduct of cellular respiration and are involved in normal signaling processes [5]; however, high levels of ROS can be problematic, resulting in oxidative stress and aberrant changes in protein structure and function [7]. Antioxidants act to maintain a balance within ROS levels, low enough to participate in the normal signaling process and not too high as to cause oxidative stress. When ROS levels supersede antioxidant capabilities, oxidative stress occurs and ROS molecules alter proteins and other elements in their surrounding environment, causing disruptions in signaling and function. As high levels of ROS have been implicated in disease development and/or progression, increasing antioxidant activity may slow or prevent VaD symptoms [2].

In addition to high levels of ROS, damage to three key targets at different levels of organization in the brain may upset ROS homeostasis, leading to the cognitive decline that characterizes VaD: these are the mitochondria, endothelial cells, and the vessel walls of the cerebrovasculature [8]. While not the only sources of ROS, when any of these areas no longer function properly oxidative stress in the brain increases. Furthermore, targeting these particular areas leads to VaD or its amelioration. Glial cells also contribute to ROS levels in the brain and in all probability play a role in VaD; however, because studies showing that an increase in or the attenuation of ROS produced from glial cells has a significant effect on VaD have yet to emerge, the degree of importance that these cells carry is still up for debate. Whether damage to the mitochondria, endothelial cells, or vasculature occurs before or after ROS accumulation is also unclear and may vary depending on the causal origin of VaD. Nonetheless, targeting ROS in these three primary target locations may provide one of the first VaD-specific therapeutics. In this review, we will discuss how specific ROS impact VaD, the damage ROS cause during disease progression, and how ROS may act as a potential therapeutic target for VaD.

## 2. Vascular Dementia and Oxidative Stress

VaD refers to the progressive cognitive decline that results from a chronic inability of the cerebrovasculature to properly supply blood to the brain. A multitude of risk factors and insults—rupture, stenosis, occlusion, or narrowing of the blood vessels—can all lead to a decrease in blood flow and an ischemic state. In an ischemic environment, ischemic lesions and neuronal loss can occur, as toxins are not removed and necessary nutrition is not provided. If proper blood flow is not reestablished and an ischemic state becomes chronic, physiological damage to the brain can lead to cognitive losses. In fact, common VaD symptomology includes white-matter damage, ischemic lesions, neuronal loss, and cognitive decline [1]. VaD is currently the second most common type of dementia, accounting for nearly 30% of all dementia cases compared to Alzheimer’s disease’s 60% [1,9]; however, this prevalence may be skewed since VaD is often confused for Alzheimer’s disease [10] due to its similar symptomology and it affecting the same population of elderly adults (>65 years) [11]. Despite its widespread prevalence and dementia cases expected to affect over 150 million people globally by 2050 [12], no licensed treatments targeting the underlying etiology of VaD currently exist.

**Figure 1:**
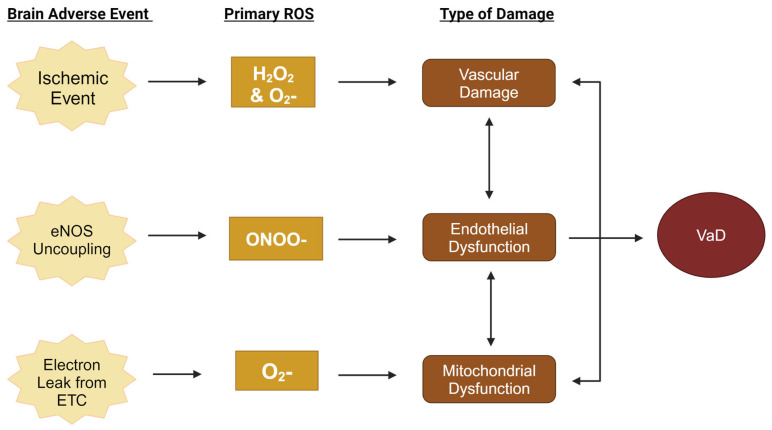
Overview of the possible mechanistic link of primary reactive oxygen species in the development of vascular dementia adverse events in the brain (stroke, eNOS uncoupling, and electron leak from the ETC) which lead to the production of ROS that contribute to much of the common pathological damage seen in vascular dementia. eNOS; endothelial nitric oxide synthase. ETC; electron transport chain. H_2_O_2_; hydrogen peroxide. ONOO−; peroxynitrite. O_2_−; superoxide. VaD; vascular dementia.

Fortunately, there are three primary targets at different levels of organization in the brain of therapeutic interest that may lead to the first VaD-specific treatment: mitochondria at the subcellular level, endothelial cells at the cellular level, and the cerebrovasculature at the tissue level often lead to VaD when damaged [13,14,15]. The relationship between each of these targets when severe enough damage occurs and how they can lead to VaD will be discussed in more detail in later sections. Briefly, vascular dysfunction is characterized by improper cerebral blood flow, which can result from instances such as blood vessel blockage or improper narrowing of the blood vessels. Endothelial dysfunction is characterized by cells’ inability to produce nitric oxide [16], whose reduced production impairs endothelial-related functions, such as vasodilation. Mitochondrial dysfunction occurs when mitochondrial enzymes no longer function properly, leading to a decrease in ATP and an increase in ROS production [17]. When mitochondria, endothelial cells, or vessels become damaged, the ongoing balance between oxidation and reduction at these sites becomes disrupted, leading to oxidative stress that turns these locations into hotspots of continuous ROS generation. While the generation of any ROS molecule tends to promote the production of others, damage that happens in different locations, in different environments, affects the composition of ROS that are produced (Figure 1).

Mitochondrial dysfunction starts in the mitochondria, where protons are stored in the intermembrane space, creating a high potential energy gradient to be used in the production of ATP. Therefore, when disruptions in the electron transport chain cause the destructive release of that potential energy in the form of the increased leakage of electrons in the mitochondrial environment, ROS such as hydrogen peroxide (H_2_O_2_) can be readily formed. Endothelial dysfunction starts in endothelial cells, where the signaling molecule nitrogen oxide (NO) is readily produced. Thus, when endothelial cells are damaged, peroxynitrite (ONOO−) is easily formed. Vascular dysfunction occurs when the blood vessels are damaged. Here, damaged blood vessels lead to the easy formation and spread of superoxide (O_2_−). While antioxidants act to decrease ROS levels, due to the selective permeability of the blood–brain barrier (BBB), the brain is limited in how quickly it can adjust its antioxidant activity when oxidative-stress-related damage does occur, as the assistance it can receive from the periphery is highly regulated. The brain is also a location with a high rate of glycolysis and already produces a large amount of ROS as a result. The combination of limited antioxidant support and a high production of ROS make the brain more susceptible to oxidative damage [18]. While a central cause for VaD has yet to be identified, many current theories implicate ROS and oxidative stress as having prominent roles. While all three biological dysfunctions mentioned eventually lead to one another, damage to these different primary targets accumulates varying amounts of different ROS types. Therefore, determining ROS composition may help in furthering understanding disease progression and inform the development of more targeted treatments for the different subclassifications of VaD.

## 3. Antioxidant Activity of Repurposed Treatments for the Management of Vascular Dementia

At the time of writing this review, no pharmacological therapies are FDA-approved explicitly for the treatment of VaD [19]. However, several Alzheimer’s disease (AD)-specific drugs have been repurposed as off-label therapeutics for the symptomatic treatment and management of VaD: donepezil [20], galantamine [21], and memantine [22]. AD is another form of dementia characterized by the accumulation of plaque and neurofibrillary tangles in the brain. This approach of using off-label AD drugs for VaD is based on clinical observations that nearly half (45.8%) of older persons diagnosed with dementia display a mixed pathology upon autopsy [23]. Patients with dementia that show pathological signs that correspond with both AD and VaD are now categorized as either AD with vascular contributions or mixed dementia, where signs of infarct (VaD), white-matter damage (VaD), and amyloid beta plaque buildup are prominent (AD).

Many of the drugs used as off-label medications for VaD to treat patients with a suspected mixed pathology include cholinesterase inhibitors, such as donepezil and galantamine. In AD, Aβ (amyloid beta) plaques and neurofibrillary tangles specifically target cholinergic neurons by binding with high affinity and specificity to the alpha-7-nicotinic acetylcholine receptor; this targeted binding mechanically disrupts the neurovascular unit by disrupting neurons and synapses [24]. Damaging cholinergic neurons and synapses preferentially impacts neocortical and limbic networks critical for judgment, memory, and awareness, eventually leading to the manifestation of the hallmark clinical features of AD. By administering drugs that can increase bioavailable acetylcholine, the proper agonist can outcompete AD plaques and tangles for receptor binding in terms of sheer number, thus preserving more functional receptors. Aβ plaques have also been shown to cause oxidative stress, alluding to their importance in disease progression [4]; however, the timing and importance of oxidative stress compared to Aβ in AD are still being debated [25]. On the other hand, the early occurrence and importance of oxidative stress to VaD are clearer, as several risk factors of VaD include oxidative stress [4]. Patients with VaD even display significantly higher signs of oxidative damage than patients with AD [26]. Thus, the efficacy of donepezil and galantamine for VaD may be in part due to their antioxidant capability.

Memantine is also indicated for AD but has demonstrated significant clinical efficacy off-label for treating VaD of a suspected mixed pathology, both alone and in combination with acetylcholine esterase inhibitors [27]. Memantine is designed to specifically target *N*-methyl-d-aspartate (NMDA) glutamate receptors in neurons and other cell types (e.g., endothelial cells and glial cells), functioning in a neuroprotective role by blocking stimulatory glutamatergic activation [22]. The overactivation of NMDA receptors by glutamate is thought to contribute to neuronal damage and cell death through excitotoxicity caused by an AD pathology. In addition, NMDA activation is linked with the accumulation of phosphorylated tau plaques, which also contributes to the progression of an AD neuropathology [28]. Because of this, memantine is commonly administered to reduce the progression of further cognitive decline by inhibiting neuronal death.

It is important to note that although beneficial for symptom management in some patients, donepezil, galantamine, and memantine cannot prevent cognitive decline in AD or VaD, but instead may slow disease progression. Additionally, these drugs should also be used with caution in patients with heart conditions, as usage can result in negative chronotropic effects: QT prolongation, bradycardia, and low blood pressure [29]. Reductions in heart rate when treating a patient with dementia may lead to syncope, falls, fractures, and hospitalization. Moreover, as heart conditions are often a comorbidity associated with VaD, it should come as no surprise that acetylcholine-targeted AD medications have a lower efficacy rate than when used for AD patients. The side effects of these same medications are substantial enough that current clinical guidelines recommend easing a patient into the medication, “starting low and going slow” with dosage [30].

Antioxidants and nutritional additions have been considered as ways of improving current treatment options for dementia. Several studies have shown that each of the currently used off-label drugs display some antioxidant activity; therefore, some potential exists for each to contribute, to varying degrees, to VaD prophylaxis by scavenging ROS. Table 1 was compiled to compare the ROS scavenging ability of a few of the most common repurposed AD medications used to treat VaD with several common antioxidant compounds via a standardized bench assay utilizing 1,1-diphenyl-2-picrylhydrazine (DPPH) to assess antioxidant activity in vitro. While all of the listed repurposed medications for VaD do display some amount of antioxidant activity, none are as powerful at scavenging ROS as “pure” antioxidants; however, simply having strong antioxidant capability may not be enough to see the amelioration of VaD symptoms. As alluded to with memantine, although its antioxidant capability is not as strong as that of donepezil or galantamine, its higher efficacy in VaD may be due to its activity towards ROS in key locations with NMDA receptors. NMDA receptors are involved in NOX-produced ROS [31], a source of oxidative stress that leads to VaD, discussed in further detail in a later section.

## 4. Importance of ROS in Alzheimer’s Diseases and Vascular Dementia

ROS as a therapeutic target for dementia has been well explored in AD with varying results. The endogenous expression of antioxidants in AD patients is low and oxidative stress increases Aβ production (one of the hallmarks of AD) [36,37]; thus, increasing antioxidant activity as a therapeutic seems like a logical avenue to explore. However, clinical trials that focus on the restoration of antioxidant activity as a treatment for AD have largely reported a decrease in oxidative stress, with no improvement in cognition and/or minimal changes in other signs of dementia progression [38]. The lack of a therapeutic effect on cognition in these clinical trials may indicate a critical period where the damage caused by free radicals is too vast for antioxidant intervention alone to be effective. Alternatively, the failure of antioxidant-based clinical trials for AD may indicate that ROS buildup is more of a downstream consequence of plaque accumulation and neuronal death, rather than oxidative stress being a causative element in of itself. During the progression of AD, ROS likely do not become a problem until after the formation of plaques and the death of neurons. On the other hand, because oxidative damage occurs early on or immediately after an ischemic event in VaD [39,40], ROS might be more heavily involved in disease onset and progression [3].

Having different casual elements likely contributes to the differences in the progression of cognitive decline that occur during VaD and AD [41,42]. The loss of cognition in VaD progresses in a stair-like manner, as opposed to AD, which progresses in a continuous manner [42,43]. This descending stair-like pattern of decline purported in VaD is more in line with a compensatory increase in endogenous antioxidant activity that fails to adequately resolve oxidative stress within the environment. Antioxidants respond to oxidative stress in a reactive and predictive manner [44]. In this interpretation, a plateau, or no change in cognitive function, represents a time when reactive antioxidant activity is actively working to decrease ROS levels, maintain ROS equilibrium, and prevent oxidative-stress-associated neuronal damage. The vertical drop, or a decrease in cognition, represents a time when antioxidant activity fails to maintain appropriate ROS levels as oxidative stress begins to build. This increase in oxidative stress then leads to neuronal and cognitive loss. In response to these losses, antioxidant levels increase and the cycle renews. The inability of antioxidants to maintain ROS levels is well represented in this stair-like pattern of cognitive decline and highlights the importance of oxidative stress in VaD. However, there are many ROS types and each ROS molecule interacts with its environment differently. Additionally, the composition of ROS molecules produced differs depending on where the ischemic event originates. As diagnosing practices for VaD can still be problematic [45,46], the identification of the ROS molecule driving this stair-like change in cognition may serve to better standardize current practices.

## 5. Primary Reactive Oxygen Species in the Pathogenesis of Vascular Dementia

### 5.1. Low-Density Lipoprotein, Hydrogen Peroxide, and the Mitochondria

Oxidative stress can act as a constant source of cellular damage, which can be increased by exposure to ionizing radiation, cigarette smoke, or high concentrations of sugar. ROS can also be produced internally from glycolysis, the pentose phosphate pathway, and the citric acid cycle in the mitochondria. Approximately 90% of all ROS originate from the mitochondria [47]. ROS arise as byproducts of energy production in the mitochondria via the electron transport chain, which leaks electrons primarily through inefficient transfer of complexes I and III. These electrons escape to oxidize diatomic oxygen, causing the formation of superoxide, a highly reactive species. Superoxide may then directly oxidize low-density lipoprotein (LDL) or react with protons to form hydrogen peroxide (H_2_O_2_), or react with nitric oxide to form peroxynitrite [48]. While both hydrogen peroxide and peroxynitrite go on to oxidize other biomolecules and disrupt important chemical pathways, hydrogen peroxide is of special interest because of the potential to disrupt the many healthy functions it provides at physiological concentrations as a ubiquitous endogenous signaling molecule.

Hydrogen peroxide, while less reactive than superoxide, still holds strong potential to further react with and oxidize other molecules. It can be produced from superoxide spontaneously or in a reaction catalyzed by the mitochondrial enzyme superoxide dismutase. While cells produce a normal amount of hydrogen peroxide necessary for intracellular signaling, cell cycle signaling, and controlling several varieties of cell death [47,48,49], higher concentrations can cause harm in a variety of ways. Harmful levels of H_2_O_2_ can directly oxidize proteins into dysfunctional forms, oxidize lipids in cellular membranes, oxidize DNA, leading to strand breaks, and induce apoptosis. H_2_O_2_ can also react with heavy metals, such as copper and zinc, to form radical anions in the form of reduced hydroxyl groups via the Fenton reaction. It can induce angiogenesis by upregulating vascular endothelial growth factors, produce more ROS by promoting eNOS (endothelial nitric oxide synthase) through the upregulation of the enzyme itself, and facilitate leukocyte adhesion by upregulating ICAM and VCAM, among other adhesion factors. Lastly, it can also kickstart a vicious positive feedback cycle by upregulating NF-κB, which produces even more ROS while promoting inflammatory signaling cascades.

**Figure 2:**
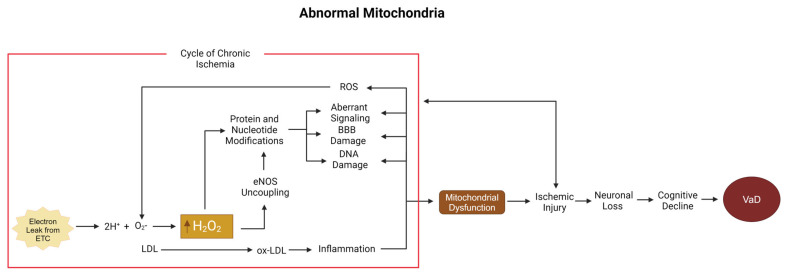
Summary of how brain-mitochondrial-sourced hydrogen peroxide contributions to vascular dementia electron leakage from the electron transport chain (ETC) in the presence of superoxide (O_2_−) lead to the production of hydrogen peroxide (H_2_O_2_). H_2_O_2_ can then modify protein and nucleotide structures, leading to various damages. H_2_O_2_ can also oxidize LDL, leading to increases in inflammation. The combined effects of high levels of H_2_O_2_ promote mitochondrial dysfunction and ultimately result in VaD.

Much of the underlying pathophysiological mechanism of VaD mimics the same roots of cardiovascular disease in other parts of the body, with manifold sequelae branching off as athero- and arteriosclerosis [8]. As in cardiovascular disease, where the buildup of arteriosclerotic plaques narrows arteries and impairs blood flow to the muscles and nerves that power the heart, VaD comprises all the downstream neurological sequelae of cerebrovascular disease, impairing blood flow and thereby oxygen transport to the brain. While dyslipidemia has emerged as a leading candidate for an underlying cause of athero- and arteriosclerosis because of the cholesterol content of the implicated sclerotic plaques, the simple presence of cholesterol alone does not lead to plaque buildup. Instead, plaque formation is inextricably linked with the formation of ROS (Figure 2).

In VaD, mitochondrial-produced ROS are a likely source involved in plaque formation. Before plaque buildup occurs, cholesterol must travel through one of several transport proteins, such as LDL. Despite LDL’s complex reputation, it is important in maintaining the structure and function of the BBB. Only following oxidation via exposure to ROS does a healthy LDL particle transform into its harmful cousin, oxidated LDL (ox-LDL). The transformation of LDL into ox-LDL can also be prompted by smoking, diabetes, and hypertension, which all increase ROS levels.

ROS and the oxidation of LDL are pivotal steps in the switch from the normal process of membrane maintenance to pathological changes, resulting in the deleterious effects of cardiovascular and cerebrovascular disease. The combined forces of such oxidation are termed oxidative stress [41,42,43]. Interactions with ox-LDL can lead to decreases in myogenic tone [50], impairing the body’s ability to respond to the ischemic state seen in VaD and the disruption of BBB integrity through tight junctions like VCAM-1 [17,51], allowing for the invasion of monocytes into the innermost fibrous layer of an artery, the tunica intima. Within the tunica intima, large monocytes differentiate into smaller macrophages, which inactivate ox-LDL via endocytosis, consuming as many particles as they can until they hit their limit and reach quiescence in the form of foam cells. As the plaque grows, neovascularization brings oxygen to its expanding mass, much like it does in a tumor, keeping the hazardous material contained until the plaque grows too large. When microvessels can no longer supply the deepest part of the plaque, its core becomes avascular, undergoing necrosis [52,53]. During this necrotic process, foam cells can undergo any of several varieties of cell death, including apoptosis, autophagy, necroptosis, and pyroptosis [52]. Following cell death, the physical integrity of the extracellular matrix encompassing the plaque becomes unstable, increasing the risk of rupture.

Ruptured plaque is a serious complication that can lead to many downstream consequences depending on where the mass originates and where it lodges. Peripheral ruptures may cause necrosis in extremities such as toes. Ruptures in or around vasculature directly supplying the heart can lead to myocardial infarction. Ruptures in vessels supplying the brain may cause microinfarcts characteristic of VaD, or, if large enough, may cause transient ischemic attacks or stroke, which can also turn into a form of VaD: infarct dementia. Additionally, H_2_O_2_ is known to induce mitochondrial dysfunction [54], a condition commonly found in VaD patients [55]. As H_2_O_2_ may be important towards the development of ox-LDL in VaD, lipoprotein status may be a potential biomarker [56].

### 5.2. Peroxynitrite, Nitric Oxide, and Endothelial Cells

VaD has a strong association with endothelial cells and eNOS (endothelial nitric oxide synthase) function. In a healthy functioning endothelial cell, the eNOS enzyme (primarily located in endothelial cells) produces the signaling molecule NO to regulate vascular tone, angiogenesis, and systemic blood pressure. Endothelial cells are also a vital component of the overall vasculature structure. Thus, when eNOS no longer functions properly, it is no surprise that endothelial cells are impaired in turn and adverse conditions begin to develop. In fact, eNOS levels are significantly reduced in various animal models of VaD. Bilateral common carotid artery occlusion is a surgical model used to induce VaD, which results in a decrease in eNOS levels and elevated levels of ROS [52]. Enos-deficient mice are a transgenic mouse line where the loss of one or both copies of the eNOS gene leads to the gradual development of VaD with age. Changes in ROS levels over time in eNOS-deficient mice have yet to be looked at; thus, it is unknown at what age the surgical and transgenic models overlap. High ROS and low eNOS levels are potential signs of eNOS dysfunction, a common biological disorder of VaD.

eNOS function can be negatively impacted by several factors: insufficient production, improper function, and cofactor deficits, all of which can lead to eNOS uncoupling and endothelial dysfunction. eNOS produces NO by turning L-arginine and oxygen into L-citrulline and NO through enzymatic activity bolstered by the co-factor BH_4_: L-arginine + O_2_ → L-citrulline + NO. Without dimerization or without BH_4_, eNOS uncoupling occurs, causing the enzyme to produce superoxide instead. The combination of NO from dimerized eNOS and superoxide from uncoupled eNOS within the same cell provides perfect conditions for the spontaneous formation of peroxynitrite (ONOO−). ONOO− and ROS accumulation combined with improper NO production can also lead to endothelial dysfunction [18]. eNOS uncoupling is a common sign of endothelial cell dysfunction, which has been proposed as a mechanism involved in the development of VaD [57]. eNOS uncoupling can also aggravate ischemic injuries [58]. As ischemia is a common state in VaD, eNOS uncoupling is likely important in disease progression as well. Notably, the ROS molecule of greatest concern in endothelial dysfunction is ONOO− [59,60].

**Figure 3:**
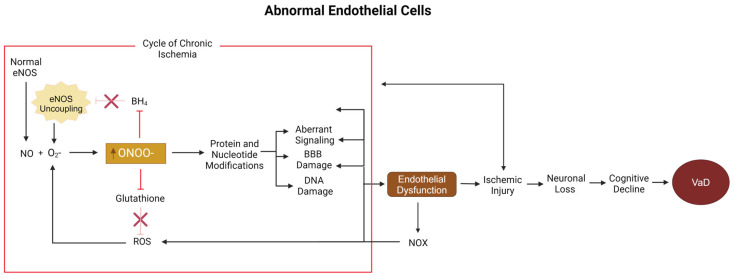
Summary of peroxynitrite contributions to vascular dementia in abnormal brain endothelial cells. The reaction between nitric oxide (NO) and superoxide (O_2_−) from eNOS uncoupling leads to the production of peroxynitrite (ONOO−). ONOO− is then able to modify neighboring structures, promote eNOS uncoupling, and inhibit glutathione, preventing its antioxidant activity. Various ONOO− induced damage causes endothelial dysfunction and increases in NOX, and ultimately leads to VaD.

ONOO− is an ROS molecule formed by the interaction between NO and O_2_− [18]. Not only is ONOO− a stronger oxidant than O_2_−, but it can also readily pass through the BBB, resulting in the easy spreading of oxidative damage [61]. ONOO− can also cause many modifications to the structure and function of enzymes [62], DNA, and RNA [62,63]. Modifications of RNA and DNA lead to the disruption of transcription and translation [38]. ONOO− can also inhibit manganese superoxide dismutase and mitochondrial enzymes, resulting in a reduction in ATP and increases in ROS levels [63]. ONOO− can also inhibit glutathione, a major antioxidant [64], decreasing the chances that it or other ROS molecules will be neutralized [39]. Therefore, not only does ONOO− act to increase its own production, but it also acts to prevent its levels from decreasing as well. ONOO− can also modify BH_4_, making it unusable as a cofactor, thereby encouraging eNOS uncoupling [62]. eNOS uncoupling can then lead to ischemic injury [61], which can cause neuronal death, white-matter damage, and increases in ROS production. In response to an ischemic injury, endothelial cells will increase NO production in an attempt to increase blood flow through vasodilation; inadvertently, this will instead increase the pool of reactants needed for superoxide to form ONOO−, creating more ROS and exacerbating an already-toxic environment [65]. In these ways, ONOO− acts to establish its own positive feedback loop and promote a state of chronic ischemia. Chronic ischemia combined with the previously mentioned damage leads to VaD (Figure 3).

Lastly, ONOO− can cause lipid peroxidation, interrupting normal function and contributing to BBB damage [61]. ONOO− modifies several components of the extracellular matrix: perlecan, collagen type 4, fibronectin, and laminin, resulting in impaired cell adhesion and barrier function [66]. ONOO− is also associated with increases in matrix metalloproteinase (MMP) activity: MMP7, MMP13, and MMP3, as shown by a study on a rodent stroke model [61]. Matrix metalloproteinases are a family of enzymes responsible for extracellular matrix breakdown. It is possible that ONOO− may be able to modify metalloproteinases to increase its activity. For instance, when ONOO− levels are artificially decreased using FeTPPs, tyrosine nitration (a marker of ONOO− modification) and MMP3 activities are significantly reduced [61]. ONOO− modified components in the BBB, as well its potential to increase the enzymatic breakdown of the BBB, will weaken BBB integrity and reduce the vasculature‘s ability to regulate what comes into and out of the brain’s environment.

In summary, NO dysregulation may increase the risk of VaD through a variety of mechanisms. First, the downregulation of NO may induce VaD by reducing vasoreactivity and increasing the average vascular tone by reducing the ability of arterioles to dilate in response to normal stimuli, leading to cognitive decline via cerebral hypoperfusion. Next, NO in the presence of ROS, such as superoxide, can react to form ONOO−, which damages endogenous antioxidants, such as glutathione, increasing overall ROS levels, leading to not only oxidative stress but also an ROS−producing positive feedback cycle. Additionally, ONOO− can lead to endothelial dysfunction, a marker that leads to the development of VaD in animal models. Lastly, ONOO− damages the BBB, further contributing to the endothelial damage and cognitive decline that define VaD. Taken together, these direct and indirect pathways to endothelial damage and an ischemic state suggest that NO must be considered a significant contributor to VaD and its characteristic cognitive decline.

### 5.3. Superoxide and Hydrogen Peroxide, NOX, and the Cerebrovasculature

The third leading cause of death in most developed countries is stroke. While there have been great advances in reduction in stroke mortality, a majority of stroke survivors develop VaD, post-stroke dementia, or mixed dementia, in addition to physical disabilities [61]. In this regard, when the reduction in cerebral blood flow and oxygen (hypoxia) persists into a state of chronicity after a stroke, extensive white-matter lesions, a well-known risk factor for VaD, can occur [13,67,68]. Even with the hypoxia associated with ischemic stroke, the loss of normal respiration causes mitochondrial respiratory chain issues, where along with the intermediates in complex I–III, they interact with any remaining oxygen and produce ROS via NOX [69]. These lesions occur not just through hypoxic cell death, but are also a product of neuroinflammation and the disruption of the glymphatic pathway’s neurorepair mechanisms [70]. White-matter lesions and cognitive decline, while due to multiple co-morbidities including age, hypertension, atherosclerosis, and metabolic syndromes, cannot be separated from the damaging effects of ROS [68,70,71,72].

The primary sources of ROS dysregulation during aging, hypoperfusion, hypertension, and stroke, are membrane-bound nicotinamide adenine dinucleotide phosphate (NAPDH) oxidase (NOX) and dual oxidase (DUOX) enzymes [73,74]. The family of NOX enzymes is a key source of ROS generation throughout the body [68,73]. Seven isoforms of NOX have been found in mammals (NOX1, NOX2, NOX3, NOX4, NOX5, DUOX1, and DUOX2), with NOX1, NOX2, and NOX4 being the most prominent mediators of ROS in the brain. While NOX is prevalent throughout multiple cell types, the effect of ROS production on the vasculature plays the largest role in VaD [75]. Within the seven isoforms previously mentioned, four isoforms—NOX1, 2, 4, and 5—are derived from vascular cells [68,76]. Within the makeup of blood vessels, there is further differentiation in terms of NOX expression: endothelial cells express NOX1, NOX4, and NOX5; adventitial fibroblasts express NOX2 and NOX4; platelet cells express NOX1 and NOX2; and vascular smooth muscle cells (VSMCs) express NOX1, NOX4, and NOX5 [74,77,78,79,80]. Due to their localization in the vasculature, NOX1, NOX4, and NOX5 play a key role in protecting and maintaining BBB integrity [49,52].

In the NOX family, NOX1 and NOX2 are the main sources of ROS [73,77]. NOX acts as a catalyst to transfer electrons from the cytosol across biological membranes and into both intracellular and extracellular compartments [73,75]. This process culminates with oxygen as the final electron acceptor, resulting in the release of superoxide from the enzyme: 2O_2_ + NADPH → 2O_2_− + NADP + H+ [68,75,81]. NOX1 ROS production is connected to multiple substances expressed via atherosclerosis, vasoactive agonists, pro-inflammatory cytokines, and atherogenic particles that cause continued NOX1-derived ROS production [68,73,74,82]. NOX2 plays a key role in neuroinflammation and the production of white-matter lesions that cause memory and tissue damage [70,79,83]. Hallmarks of early VaD, endothelial injury, and reduced NO production are tied closely with large increases in levels of NOX2 [77]. NOX2 also plays a direct role in platelet activation/thrombosis and is upregulated in patients with atherosclerosis [84], a risk factor for VaD.

**Figure 4:**
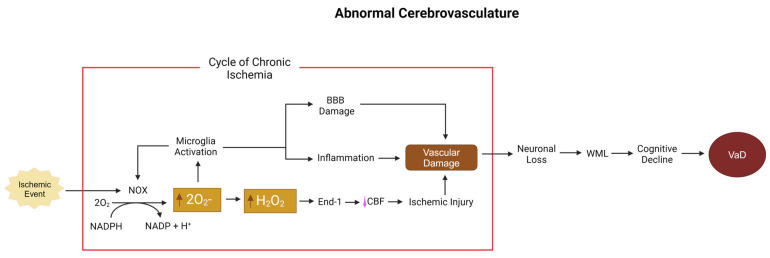
Summary of superoxide contributions to vascular dementia in abnormal cerebrovasculature. An ischemic event can lead to the upregulation of NOX enzymes and the production of superoxide (O_2_−) and hydrogen peroxide (H_2_O_2_). O_2_− can promote inflammation and BBB damage. H_2_O_2_ can prolong the ischemic state, leading to further injury. Ultimately, the damage induced by O_2_− and H_2_O_2_ leads to VaD.

NOX1 has a large role in neuroinflammation, BBB disruption, and ischemia [85,86]. There are two main signaling pathways concerning NOX1: the ATF-1-MEF2B pathway and the ERK1/2-JunB pathway, both induced by factors such as angiotensin II (Ang II), PDGF, and prostaglandin (PG) F2α [85]. In ischemia and hypoxia, the reperfusion state after blockage clearance induces NOX1 expression, furthering ischemia-associated tissue damage and BBB disruption [85]. In VSMCs, NOX1 has been shown to increase protein degradation, damage, and cell death due to high levels of ROS [87]. NOX1 expression levels are therefore variable depending on the type and region of insult. All forms of NOX produce an ROS end product during their catalytic conversion of NADPH [79]. NOX1, 2, 3, and 5 result in superoxide, which then transforms into hydrogen peroxide, while NOX4 and DUOX1/2 produce hydrogen peroxide directly [88]. This ROS formation causes multiple pathologies depending on their location. Some examples of neuropathologies mediated by NOX expression occur during AD. Specifically, the Aβ25–35 fragment increases NOX2 expression, thereby furthering superoxide release, which then causes microglial activation and damaging inflammation [89]. NOX4-derived hydrogen peroxide triggers endothelin-1, which reduces blood flow and can further promote VaD [90]. As summarized in Figure 4, ROS mediate vascular damage derived from hypoxic insult through secondary damage by disrupting the vascular homeostasis and decreasing BBB integrity by downregulating and harming tight junction complexes resulting in NOX-derived neuronal damage and endothelial dysfunction [82,91].

Compared to other members of the NOX family, where expression is inducible via insult, NOX4 is both constitutively expressed and inducible [85,87,92]. TNOX4 is also significantly more prevalent throughout the vasculature than the other NOX enzymes, and NOX4 is responsible for basal ROS production [77,82,93]. NOX4 produces hydrogen peroxide instead of the pre-form of superoxide, due to it being the constitutively active NOX isoform and oxygen sensor [94]. NADPH oxidases are therefore critical for both the homeostatic function of the cerebral vasculature in addition to the required regulation of NOX signaling, otherwise leading to pathological states causing further damage to the endothelial, microglial, and neuronal populations of the brain. Basal NOX4 is a housekeeping gene for VSMCs and presents under hypoxic vascular damage [93,95]. Inducible NOX4 occurs due to hypoxic/ischemic conditions [92]. This expression acts as a natural oxygen sensor in the lungs, with Mittal et al. reporting that NOX4 expression increased in the pulmonary arteries after chronic mild hypoxia in mice [96]. Diebold et al. also found that NOX4 is a target gene for hypoxia-inducible factor-1α (HIF-1α) and inflammatory signaling [85,92]. NOX-derived ROS at the molecular level constrict capillary vessels, for example, via NOX4 from pericytes, which results in restricted blood flow and the loss of oxygen and glucose to the brain, inducing mild hypoxia [79,90].

## 6. Potential Translational Antioxidant Therapeutics for Vascular Dementia

Antioxidants describe any molecule able to neutralize ROS, thereby preventing ROS-induced damage and oxidative stress. Antioxidants come in various forms and can be derived from various sources, thus ensuring a range of differences in how they perform their task. Antioxidants can be found endogenously, attained from one’s diet, or artificially created. Some antioxidants are enzymes capable of neutralizing multiple ROS molecules; others have no enzymatic activity and are non-reuseable, meaning that they can only neutralize one ROS molecule before losing their antioxidant function. The following section lists antioxidants that have been used as treatments in various animal models of VaD (Table 2; Section 6.1, Section 6.2, Section 6.3, Section 6.4, Section 6.5 and Section 6.6), as well as antioxidants that have been used to reverse or prevent common VaD pathologies in vitro: mitochondrial dysfunction, endothelial dysfunction, or vascular damage (Section 6.7, Section 6.8 and Section 6.9), and are prime candidates for future in vivo studies. All of the listed antioxidants hold great potential as treatments for VaD and require further research to uncover and validate their therapeutic capabilities.

### 6.1. Carotenoids

Carotenoids are lipophilic pigments [117] commonly found in plants such as carrots and tomatoes [118,119]. Carotenoids can be found in many forms, but five dietary compounds are commonly found in the human body: α-carotene, β-carotene, lutein, zeaxanthin, lycopene, and β-cryptoxanthin [117]. In contrast to other antioxidants, such as vitamin E, carotenoids are destroyed after neutralizing a single ROS target and cannot be regenerated [118]. Some carotenoids are also precursors of vitamin A [118]. While vitamin A is reduced in VaD patients, it does have antioxidant activity [120,121]; however, few representatives outside of the pro-vitamin A carotenoids (carotenoids able to be converted into vitamin A) have yet to be tested as a therapeutic for VaD.

In addition to their capabilities as a vitamin A precursor, carotenoids like lutein and zeaxanthin are able to cross the BBB [117], thus increasing their potential efficacy on the brain and making them more viable candidates for VaD therapeutics. Other carotenoids tend to aggregate in membranes, where their orientation impacts membrane fluidity, stability, permeability, and signaling [118]. In VaD patients, carotenoid levels are significantly low, while ROS levels are high [119,122]. Disruptions to the BBB by vascular lesions and ROS-induced modifications by molecules such as H_2_O_2_ in VaD may be contributing to the displacement of carotenoids, lowering carotenoid levels and activity. While not a VaD model, a study performed in a rat model of AD found that lycopene treatment significantly restored mitochondrial respiratory enzyme activities and attenuated mitochondrial oxidative stress [118]. Due to their positioning in the BBB and their ability to improve mitochondrial function, carotenoids may have a role in the maintenance and protection of not only the BBB, but of the mitochondrial membrane as well.

The usage of a different carotenoid, lycopene, in animal models of VaD has also found favorable results. Female rats used in Ning-Wi Zhu et al., 2020, that underwent the BCCAO model of VaD and were treated with lycopene showed improved cognitive performance and increased antioxidant activity. Of the dosages tested, 100 mg/kg had the greatest effect on lowering ROS levels, increasing SOD activity, and attenuating Morris water maze performance [102]. In fact, 50 and 200 mg/kg increased ROS levels, suggesting an important range of too low to be effective and too high becoming toxic. Of note, there is speculation that carotenoids may be able act as prooxidants, contributing to a higher oxidative environment, rather than decreasing it [105]. While the exact conditions that prompt this change are still being investigated, carotenoids under prooxidant conditions are believed to be chemically unstable and lead to the production of ketones and aldehyde, thus promoting direct and indirect prooxidant damage [105].

However, gerbils who were given VaD were treated with less (20 mg/kg) lycopene still showed improvements in terms of cognitive performance, increased antioxidant activity, and lowered signs of apoptosis and inflammation [103]. The different results in terms of dosage likely further implicate the common hurdles of translational research from animal models or that gerbils provided with a higher concentration of lycopene could have seen even more improvement. These two studies also suggest that lycopene may be an effective treatment in both males and females.

In a recent study examining the relationship between the concentration of carotenoids in plasma and cognitive performance in the elderly, it was observed that low plasma carotenoid levels were associated with lower cognitive functioning [119]. Furthermore, when carotenoids were combined with docosahexaenoic acid (DHA), general cognitive performance improved significantly in elderly woman [119]. DHA is an omega-3 fatty acid, commonly found in fish, with neuroprotective, anti-inflammatory, and antioxidant capabilities [123]. Clinical trials using DHA for early AD and mild cognitive impairment saw positive results [123], but no trials for VaD have been completed.

In Khian Giap Lim et al., 2022, mice treated with 50 mg/kg or 100 mg/kg of beta carotene in an induced diabetic VaD model showed improved cognitive performance and decreased acetylcholinesterase levels compared to their untreated demented counterparts [101]. The beta-carotene-treated mice in this study also had increased levels of glutathione (GSH) and lower levels of thiobarbituric-acid-reactive substances, making them more biochemically similar to the control group [101]. One dose did not seem to be better than the other in terms of the measured parameters; however, the decrease in acetylcholinesterase levels found with beta-carotene is similar to that of current off-label drugs used for VaD. Interestingly, beta-carotene-treated mice in this study performed just as well as donepezil-treated mice in all experiments performed in this study. Perhaps changes in administration methods of beta-carotene, such as dosage or route, could outperform donepezil.

Treatment with lutein at different dosages (0.5 mg/kg and 5 mg/kg) in BCCAO male rats showed positive, but little, difference between the two treatment groups. Both lutein-treated groups were able to display attenuated performance in terms of a Morris water maze and antioxidant activity. Furthermore, treated mice also had increased neuronal connectivity and a higher population of pyramidal neurons in the CA1 region [104]. The one reported difference between the two dosages was that 5 mg/kg showed a slight improvement in the passive avoidance test [105]. Lastly, as opposed to the previous carotenoids, in a comparison of three different dosages (50, 100, and 200 mg/kg) of astaxanthin-treated UCCAO mice, improvement in cognition, antioxidant levels, and decreased levels of IL4 was shown in a dose-dependent manner [105].

Carotenoids have consistently been shown to be able to improve cognition, reduce ROS, and increase antioxidant activity in animal models of VaD. Lycopene has even been shown to work in both female and male animal models; however, the possibility of prooxidant conversion and the effective range of dosages of different carotenoids highlight the importance of careful planning.

### 6.2. Idebenone

Mitochondrial complex I is highly susceptible to impairment in conditions of oxidative stress and is dysfunctional in several neurological diseases [124]. Complex I moves electrons to coenzyme Q10 (CoQ10) and protons out of the matrix [124]. CoQ10 also happens to be an antioxidant, but displays such poor bioavailability that its potential as a therapeutic is low [124]. Another limitation of CoQ10 efficacy lies in the fact that maintenance of the coenzyme in its reduced antioxidant form (termed ubiquinol) requires an intact electron transport chain, which is impaired in AD [125]. While it is still being researched, there is some evidence of impairment in mitochondrial function in VaD as well [126]. To overcome the limitations of CoQ10, analogs such as idebenone have been constructed [38]. Idebenone is a synthetic antioxidant with higher bioavailability and is able to cross the BBB [38,124], allowing it to have a higher therapeutic potential than CoQ10. Idebenone has been shown to be neuroprotective in conditions of oxidative stress [124]. In a rat model of VaD using BCCAO, animals treated with idebenone had attenuated levels of hippocampal CA1 neurons, along with attenuated Morris water maze performance [106]. Idebenone has not been explored as a therapeutic in VaD as extensively in animal models as carotenoids have, warranting more studies. Despite this, idebenone has been used in clinical trials with positive results, more of which will be discussed later in this review [127].

### 6.3. Alpha-Lipoic Acid

Alpa-lipoic acid (ALA), also known as thiotic acid, is an endogenous antioxidant [37] found in the mitochondria. Mitochondria are an important site of energy metabolism. One enzyme involved in glucose metabolism is the pyruvate dehydrogenase complex, shuttling Acetyl-CoA into the tricarboxylic acid cycle. Pyruvate dehydrogenase complex activity is reduced in patients with VaD but can be stimulated by ALA [97]. Pyruvate dehydrogenase uses ALA as a cofactor [12]. SDH activity is relatively unaffected in patients with VaD compared to non-demented patients [97]. ALA also induces the transcription factor Nrf2, which regulates several different antioxidant enzymes [12]. As an endogenous mitochondrial enzyme, ALA is likely one of the first enzymes affected by mitochondrial dysfunction. Thus, increasing ALA levels should help prevent further damage from mitochondrial dysfunction in VaD.

In an animal model of VaD, BCCAO rats treated with ALA showed decreased ROS levels, improved cognition, and attenuated antioxidant activity in the hippocampus [99]. Additionally, acetylcholine levels increased, while acetylcholine esterase levels decreased [99]. Like the carotenoid beta-carotene, its ability to increase acetylcholine levels while displaying antioxidant activity is similar to that of off-label drugs used to treat VaD. As oxidative stress is important in the development of VaD, ALA and beta-carotene may display a stronger therapeutic effect than current VaD treatments if their antioxidant activity is higher.

### 6.4. Resveratrol

Resveratrol (3, 5, 4′-trihydroxystilbene or RSVL) is a polyphenol commonly found in grapes and red wine [128]. RSVL is believed to have antioxidant, anti-inflammatory, and neuroprotective effects [98]. RSVL is also a vasodilator, inducer of eNOS (contributing to increases in NO production), antithrombotic, able to decrease cholesterol carried in lipoprotein fractions, and induces SIRT1 [129]. Sirtuin (SIRT) 1 is part of a family of NAD-dependent protein deacetylases involved in mitochondrial function, self-renewal, and neuroprotection [128]. SIRT1 inhibits NADPH oxidase activation and protects endothelial function, thus complicating the link between resveratrol and cerebrovascular endothelial function [128].

In a diabetes-induced model of VaD, rats treated with RSVL showed improvement in cognition and molecular markers: TNF-α, eNOS, IL-1B, and HO-1 [98]. Results from this same study even showed that measurements of body weight, blood glucose, carbachol levels were all restored to baseline levels in the RSVL-treated group [98]. While no changes in SNP were found [98], cognition still improved, suggesting a more complicated process involved in preventing neuronal loss or increasing neurite growth.

RSVL improves cognitive function, reduces MDA, increases SOD, and decreases hippocampal apoptosis in BCCAO rats [74]. In a rat model of VaD through permanent BCCAO, the administration of RSVL improved learning and memory function in treated animals [107,128]. RSVL administration has also been shown to reduce levels of malonyldialdehyde, a marker of oxidative stress in neurodegenerative disease, in the cerebral cortex and hippocampus, and increased superoxide dismutase activity as well as GSH levels [107,130]. RSVL-treated VaD rats also show a reduction in apoptosis [100], lower ROS levels [108], and increased synaptic plasticity [109,112]. Furthermore, RSVL treatment has shown a reduction in GFAP [110] and effects on the Akt/mTOR pathway [111]. Male wistar rats with BCCAO-induced VaD given 20 mg/kg of RSVL, as opposed to 10 mg/kg, showed greater improvement in cognition as well as antioxidant levels, and had lower acetylcholinesterase activity as well as nitrite levels. With the exception of BDNF levels, where 10 mg/kg showed higher levels, the higher dosage of RSVL seems to have a greater therapeutic effect [113]. Overall, RSVL shows great potential as a VaD therapeutic. Not only does it act similarly to current treatments used for VaD, but it also increases synaptic plasticity. The positive effects on neuronal growth that RSVL displays may be the key to developing a VaD-specific therapeutic.

### 6.5. Selenium

Selenium is a trace element that regulates the activity of the antioxidant system in the brain: glutathione peroxidase, thioredoxin reductase, and methionine sulfoxide reductase [131] in the form of selenoproteins [114]. Selenium and selenium-containing compounds have strong antioxidant activity against ONOO− [131]. Several selenium containing compounds are better ONOO− scavengers than GSH [132]. The inhibition of NO production will also prevent ONOO− formation, but it will interfere with numerous other NO-mediated physiologically relevant processes [133]. In a rat model of VaD, selenium treatment resulted in attenuated cognition, improved blood flow in the posterior cerebral artery, increased antioxidant activity, and increased synaptic plasticity [114]. Due to the synthetic nature of selenium, work can be carried out to modify it to improve its efficacy, bioavailability, or antioxidant activity.

### 6.6. Curcumin

Curcumin is a flavonoid with antioxidant and anti-inflammatory capabilities found in turmeric [115,134]. Curcumin is unstable and easily degradable [115,134], therefore limiting its bioavailability. The therapeutic usage of curcumin has been successfully improved by combining it with other compounds [134]; however, mixing other compounds with curcumin may conflate its antioxidant activity and further complicate its potential as a therapeutic. Luckily, Runfang Zhang et al., 2021, developed Cur20, a modified form of curcumin with greater stability, while maintaining antioxidant activity [115].

Runfang Zhang et al., 2021, also tested their new construct against curcumin in a UCCAO mouse model of VaD. Their study showed that while curcumin worked well to reverse both the cognitive and chemical changes associated with VaD, Cur20 trended better in many tested parameters. Cur20-treated mice showed better cognitive results than curcumin-treated mice in the MWM test and the better attenuation of SOD levels. Although curcumin had the greatest effect on lowering ROS and MDA, their levels were still significantly low in Cur20 as well. Additionally, Hif1α levels were higher in Cur20, but curcumin-treated animals showed levels closer to those of control animals. Cur20 has a lot of potential as a therapeutic for VaD. Nonetheless, more research on the effects of Cur20 is warranted. The effectiveness of Cur20 in females is still unknown. Moreover, Runfang Zhang et al., 2021, did not report any attenuation of brain physiology and only performed one cognitive test.

Another point of contention in discovering therapeutics is dosage. In fact, a study by Yaling Zheng et al., 2021, using a higher dosage showed that curcumin also had beneficial effects [116]. Curcumin-treated animals showed the attenuation of neuronal loss in the hippocampus and a decrease in various inflammatory markers (IL1β, IL6, and TNFα) and apoptotic markers (NFkb, Bax, and cleaved caspase 3). Taken together, these two studies show that curcumin is worth exploring further as a therapeutic for VaD. Moreover, clinical trials for other ailments have shown that curcumin is well tolerated [135].

### 6.7. Endothelial-Cell-Targted Antioxidants

Endothelial dysfunction and ONOO− can be targeted through the use of antioxidants. Vitamin D is an antioxidant whose deficiency has been associated with endothelial dysfunction [136,137,138] and increased VaD risk [139]. Vitamin D deficiency is also associated with increased oxidative stress and reduced antioxidant activity [140]. As discussed throughout this paper, increased ROS, endothelial dysfunction, and decreased antioxidant activity are highly indicative of VaD. Fortunately, supplementation has been shown to reverse these deleterious effects in in vitro and in vivo models [140]. Vitamin D is unique in that levels in the body can be increased exogenously, by simple exposure to the sun or sourced from food such as fish or mushrooms [141]. Therefore, there are multiple avenues in which the bioavailability of vitamin D can be manipulated for therapeutic use.

Another antioxidant that can be sourced from food is hesperitin. Hesperitin is a type of flavonoid attained from citrus fruit and tomatoes [142]. Hesperitin increases NO production from endothelial cells [143] and inhibits oxidative-stress-induced neuronal apoptosis [144]. In a randomized, parallel, double-blind clinical trial of participants with stage 1 hypertension, patients who consumed hesperitin-enriched orange juice every day for 12 weeks had increased levels of ischemic reactive hyperemia and reduced IL6 levels [145]. As hesperitin is able to cross the BBB [142] and restore blood flow while reducing inflammation in the periphery, it could be useful for the brain as well. Additionally, hesperitin may be able to prevent or reverse endothelial dysfunction through its promotion of endothelial function and inhibition of inflammation and BBB rearrangement, detailed in a review by Maria Imperatrice et al., 2022 [143]. As it has already been used in clinical trials, hesperitin is safe to use and well tolerated in humans. Furthermore, combined with its ability to decrease ROS, inflammation, and neuronal ROS, while increasing NO, it is a wonderful candidate for VaD treatment.

A synthetic antioxidant option to compete with selenium are metalloporphyrins. Metalloporphyrins are porphyrin rings with a metal ion in their center [146]. Porphyrin rings are naturally occurring structures found in hemoglobin and in plants, aiding in photosynthesis [146]. Depending on the type of metal a metalloporphyrin contains, its abilities and characteristics can easily be altered [146]. Some metalloporphyrins have strong scavenging abilities, particularly for ONOO− [62]. Due to their customizability, more research into the activity of each metalloporphyrin created is needed. Lastly, zingerone is an antioxidant compound found in ginger root and other spice plants [147]. Zingerone is effective at scavenging ONOO and its precursors: O_2_− and NO− [147]. It also reduces tyrosine nitration, a marker of ONOO modification [147]. As zingerone is able to attenuate all aspects of ONOO−, formation, creation, and modifications, it has great therapeutic potential for VaD, where endothelial dysfunction and ONOO− are importnat. Furthermore, zingerone is a plant-based antioxidant and already consumed in the human diet, so it will likely be well tolerated in a clinical trial as well.

### 6.8. Mitochondria-Targeted Antioxidants

Besides idebenoone, other synthetic CoQ10 analogs have been developed. MitoQ, MitoVitE, MitoPBN, MitoPeroxidase, and amino-acid-based tetrapeptides are mitochondrially targeted antioxidants that have been formulated to cross the BBB and infiltrate the mitochondria [12,37,125]. These targets are known to quickly counteract free radicals in mitochondria and diminish the toxicity levels [37]. Because idebenone has shown such promising effects in animal models and patients with VaD, comparing it to the other CoQ10 analogs may provide useful alternatives when investigating the details of therapeutic effect: dosage, administration route, etc.

Of the CoQ10 analogs listed in this section, MitoQ has been very popular and has been shown to reduce oxidative damage while helping to regulate mitochondrial function [12]. MitoQ can function independently of a functioning electron transport chain [125]. MitoQ was able to improve age-related arterial endothelial dysfunction in mice [148]. Notably, there is also some discourse in regard to whether MitoQ does not [125] or does display prooxidant [5] activities. Similarly to carotenoids, the conditions of this prooxidant switch are unclear.

Oleocanthal and oleuropein are two important polyphenols of olives and olive oil with antioxidant activity [149]. Oleocanthal is able to prevent the oxidation of LDL [149], is the prominent factor in olive oil’s ability to lower Aβ levels, increases synaptic plasticity, and lowers signs of apoptosis [150]. Oleocanthal-rich extra virgin olive oil has been shown to increase the therapeutic effect of donepezil [151] and protect against mitochondrial dysfunction [152]; however, much of the work carried out with olive oil polyphenols in dementia has only been performed for AD.

### 6.9. Cerebrovasculature-Targeted Antioxidants

Vitamin E has been frequently studied in models of AD. Correlations made from a subset of participants from the Honolulu-Asia Aging Study found that participants diagnosed with VaD or mixed dementia that had taken both vitamin E and vitamin C performed better on cognitive tests [153]. A separate study also found that total cholesterol and vitamin E levels in blood were lower in VaD patients [154]. Nutritional deficits in beta-carotene and vitamin C have also been found to be low in patients with VaD [120,155]. As VaD is associated with multiple vitamin deficiencies, it would be interesting to see if the restoration of one or a combination of these vitamins would result in a therapeutic effect.

There are also antioxidants that can either directly or indirectly inhibit NOX2 activation, a main source of ROS in the vasculature. Bj-1301 has strong indirect antioxidant activity by blocking the method of NOX2 activation and the translocation of subunits from the cytosol to the cell membrane, thus preventing the production of ROS from these two sources [156]. While this review focuses on direct antioxidants, due to the nature of NOX2 localization, there are more indirect inhibitors with antioxidant effects. Two indirect antioxidants are rosuvastatin and atorvastatin. They work by inhibiting platelet NOX2 activation, PKC phosphorylation, and p47phox translocation, which reduce platelet ROS production [157,158]. Another indirect antioxidant is dexmedetomidine, an α-2 adrenergic receptor that inhibits the hypoxia-based NF-κB signaling in microglia, reducing NOX2 ROS production [159]. The inhibition of ROS via antioxidants is not a new concept; however, the capability of antioxidants to combat ROS and NOX-derived ROS can change drastically with route of administration, dosage, and type, which leads to difficulties in developing antioxidant therapies [73,160,161].

## 7. Clinical Trials Using Antioxidants for Vascular Dementia

Oxidative stress is one of the major etiological factors of VaD and AD [162]. The use of antioxidants for prevention or therapy has produced conflicting results due to their low permeability of the blood–brain barrier so far. Although a number of antioxidants such as vitamin E [163,164], curcumin [165], flavonoids [166], GSH [167], and ALA [168] have been evaluated for clinical trials of mild cognitive impairment and AD, only a limited number of clinical trials evaluated the potential of antioxidants in the context of VaD (Table 3).

Idebenone has enhanced solubility and pharmacokinetics and is known to prevent lipid peroxidation and mitochondrial oxidative stress [173]. Idebenone has shown its therapeutic potential in multi-infarct dementia and moderate-to-severe dementia of the Alzheimer type [174,175]. A multicentered randomized controlled, double-blinded study showed that idebenone improved memory and cognition in mild-to-moderate mental deterioration of vascular origin [127]. Since then, Qi et al. evaluated combination therapy of idebenone with butylphthalide-attenuated serum inflammatory mediators and improved vascular function as well as cognitive function [170]. Butylphthalide is an oxidative free radical scavenger, known to regulate cerebral collateral circulation and improve microcirculation in ischemic areas as well as neural cell function in the ischemic area [176]. Similarly, a retrospective observational study demonstrated that the combination therapy of idebenone with butylphthalide [169] could improve oxidative stress, serum inflammatory factors, and cognitive function in patients with vascular dementia. Huperzine A is an alkaloid isolated from the plant called huperzia serrata, and is known to cross the blood–brain barrier, ameliorate Aβ-mediated oxidative stress, and increase acetylcholine levels, thereby improving cognitive function [177,178]. Xu et al. evaluated the therapeutic potential of huperzine in 78 patients with mild-to-moderate VaD; they found that huperzine A improved the memory, language, and visual–spatial abilities of patients with VaD [171].

The severity of neurological signs correlate with monoamine levels in the CSF [179]. HVA, 5-HIAA, and MHPG levels in the CSF were all returned to baseline in patients with VaD when treated with idebenone [180]. Although ROS, antioxidant levels, and cognitive function were not measured in this study, the authors were able to show that idebenone has a beneficial effect on attenuating the deleterious effects associated with VaD. In a clinical trial of 118 participants, a comparison of idebenone treatment between VaD (chronic cerebrovascular disorders and multi-infarct dementia) and Alzheimer’s disease (aging brain and dementia of Alzheimer’s type) was analyzed [172]. They found that idebenone treatment showed greater therapeutic efficacy in patients diagnosed with vascular dementia than those with Alzheimer’s disease [172]. By the end of the study, VaD patients outperformed AD patients on all cognitive tests given, which were the Mini Mental State Examination, Hachinski Dementia Score, Hachinski Ischemic Score, Sandoz Clinical Assessment Geriatric scale, and Serial Learning Test [172]. Of the 16 ongoing and recent clinical trials reviewed by Tran Thanh Duy Linh et al., 2022, nearly half of them are listed as targeting ROS or acting to improve specific ROS-producing targets in the brain (mitochondria/endothelial cells/cerebrovasculature) affected by VaD [181].

## 8. Discussion and Future Directions

VaD occurs due to chronic ischemia within the cerebrovasculature, resulting in various kinds of brain damage: ischemic lesions, white-matter damage, neuronal loss, and cognitive decline. While details leading to these physiological changes are still being researched, oxidative stress, endothelial dysfunction, mitochondrial dysfunction, and vascular damage are clearly important to the disease. Moreover, ROS produced from these three damaged sites likely play a vital role in developing and maintaining the chronic ischemic state that leads to VaD. Currently, as there are no specific therapeutics, treatments made for AD are being repurposed and used to manage VaD symptoms to varying degrees of success. Interestingly, the off-label AD medications used for VaD also have varying antioxidant capabilities. As oxidative stress and ROS are important, determining how to increase antioxidant activity in commonly damaged areas could provide significant aid in developing the specific and targeted therapeutics that we are currently lacking for VaD. Fortunately, many antioxidants exist that may be able to meet this challenge.

Carotenoids, RSVL, ALA, CoQ10 analogs, and oleocanthal are all antioxidants that have either been shown to or designed to attenuate mitochondrial function or target ROS in the mitochondria. Dysfunctional mitochondria and prominent mitochondrial ROS H_2_O_2_ have a strong correlation with VaD and have been proposed to be involved in its progression. Mitochondrial dysfunction can even lead to eNOS dysfunction, further promoting VaD progression. Therefore, the use of mitochondrial-targeted antioxidants could prevent further damage from occurring and potentially stop the advancement of VaD-associated cognitive decline. Moreover, carotenoids and RSVL, which have already been used in clinical trials for AD, might see more success in a VaD study. RSVL and selenium have also shown promising results in animal models, lowering ROS levels and attenuating cognition. Additionally, zingerone, vitamin D, hesperttin, and metalloporphyins have proven themselves to be capable ONOO− scavengers in vitro. As animal models have shown that endothelial dysfunction alone can lead to VaD, targeting endothelial ONOO− may be a strong therapeutic avenue. Lastly, RSVL, Bj-1301, rosuvastatin, and atorvastatin reduce production of the main ROS produced from vascular damage, O_2_−, and H_2_O_2_. RSVL even reduces microglia activation as well. An ischemic event in the brain is harmful enough on its own, but if O_2_− and H_2_O_2_ are allowed to persist, neuroinflammation can occur, leading to further white-matter lesions and VaD cognitive loss. Of the few antioxidants that have made it to clinical trials for VaD, a majority have used idebenone. The promising positive results notwithstanding, longer trials with a larger sample size are necessary to truly uncover the therapeutic potential of antioxidants for VaD. Moreover, ROS accumulation during VaD can happen in several different regions of the brain. The composition of ROS molecules differs depending on where damage has occurred as well, so targeting specific prominent ROS as opposed to a general application may improve the therapeutic effect. As indicated in Section 6, there are several antioxidants worthy of further research able to target ROS in certain locations. Of note, some have also been used in clinical trials for other ischemic conditions. Identifying ROS may also contribute to the better classification and diagnosis of VaD.

Questions still remain concerning appropriate dosages, bioavailability, and pharmacological interactions when using antioxidants as therapeutics. The majority of clinical trials conducted for dementia are for AD. The scant progress with antioxidants as a therapeutic for AD may have influenced the diminutive number of VaD clinical trials we have currently; however, one must remember that these two forms of dementia comprise different pathologies. As evidenced by the varying efficacies of the current off-label use of AD medications for VaD, different treatments work differently for these two conditions. Therefore, antioxidants that do not work for AD may work for VaD and vice versa. Rather than the drug used, information from the methodologies used in past AD trials can be used to better inform future studies on VaD. One possible discrepancy of the many clinical trials looking at antioxidant therapeutics is the failure to measure the antioxidant levels of participants at the baseline [182]. As such, an unknown number of participants may display different levels of antioxidant depletion when beginning the study [182]. This variation in starting points among patients may contribute to confounding or failed results. Establishing individualized treatment dosages, allowing for everyone to reach a standardized range, may help in developing better antioxidant-based therapeutics. Another important issue is the treatment timeline, as early administration may substantially increase therapeutic effects [118]. Additionally, it is important to remember that changes in the periphery do not always align with changes in the brain. Therefore, measurements of ROS and antioxidants when taken from samples such as the CSF will provide a more accurate picture of changes occurring in the brain.

A form of VaD not mentioned in detail in this study is CADASIL (cerebral autosomal dominant arteriopathy with subcortical infarcts and leukoencephalopathy) and CARASIL (cerebral autosomal recessive arteriopathy with subcortical infarcts and leukoencephalopathy). CADASIL and CARASIL are rare genetic forms of VaD caused by a mutation in the *NOTCH3* gene resulting in various physiologies: lacunar and micro-infarcts, white-matter lesions, migraine, and transient ischemic attacks [8,11,183]. The *NOTCH3* gene codes for a large transmembrane receptor in vascular smooth muscle cells [11]. Thus, antioxidant treatments as proposed in this review will likely only treat the symptoms and act as long-term medication at best, without addressing the underlying issue. As *NOTCH3* is the main culprit in CADASIL and CARASIL, treatments targeting the gene itself or other notable proteins along the pathway would be ideal.

Further research into the particulars is needed to elucidate the degree to which specific ROS molecules contribute to the development and progression of VaD. Further research into the specific capabilities of antioxidants, and which ROS type they are most effective against, will also benefit therapeutic development for VaD. Given that increases in oxidative stress are a normal part of aging and have been associated with other neurodegenerative diseases, the need for a validated system for quantifying abnormal ROS buildup in an age-adjusted manner as a key biomarker for VaD would also be a valuable diagnostic aid.

## Figures and Tables

**Table 1:** In vitro comparison of the antioxidant activity of Alzheimer’s disease medications repurposed as off-label treatments for the management of vascular dementia and antioxidant vitamins and supplements (*).

	Compound	Mechanism of Action	Effect	Side Effects	Inhibition % (DPPH)	Concentration Range (µg/mL)	Bioavailability	References
**Repurposed** **Medications**	Donepezil	AChEi	Mental clarity	Nausea, vomiting, and QT prolongation	33.5–42.3%	10–1000	Moderate (40–100%)	Vinay Munishamappa et al., 2018 [32]
	Galantamine	AChEi	Mental clarity	Dry mouth, constipation, and QT prolongation	20–35%	50–200	High (90%)	Victor Wagner Barajas-Carrillo et al., 2020 [33]
	Memantine	NMDAr antagonist	Neuronal protection	Dizziness, headache	1–4%	100–1000	High (100%)	I.G. Stankova et al., 2020 [34]
**Antioxidant Vitamins and** Supplements *	Curcumin *	Antioxidant/Anti-inflammatory	Anti-inflammatory	Gastrointestinal upset	65–75%	100–500	Very low (<1%)	Moeka Yamauchi et al., 2024 [35]
	Vitamin C *	Antioxidant	Immune support	Diarrhea (high doses)	90%	50–100	High (70–90%)	Moeka Yamauchi et al., 2024 [35]
	Vitamin E *	Antioxidant	Skin health	Bleeding risk (high doses)	70–80%	100–200	Low (20–40%)	Moeka Yamauchi et al., 2024 [35]
	Glutathione *	Antioxidant/Detoxifier	Detoxification	Rare (allergic reactions)	50–60%	100–500	Low (poor oral bioavailability)	Moeka Yamauchi et al., 2024 [35]
	Resveratrol *	Antioxidant	Cardiovascular health	Gastrointestinal upset	60–70%	100–200	Very low (<1%)	Moeka Yamauchi et al., 2024 [35]

Note: Vitamins and supplements with antioxidant effects included for comparison, denoted with asterisks (*).

**Table 2:** Antioxidant effects in models of vascular dementia.

Species	VaD Model	Antioxidant	Dose	Results		Reference
				Increase	Decrease	
Human	VaD brain tissue	(r)-ALA	1 µM, 10 µM, 100 µM, 1 mM, and 10 mM	PDHc activity (10 µm) SDH activity (1 mM)	PDHc activity (10 mM) SDH activity (10 mM)	L. Frolich et al., 2004 [97]
Human	VaD brain tissue	(s)-ALA	1 µM, 10 µM, 100 µM, 1 mM, and 10 mM	SDH activity (1 mM)	PDHc activity (10 mM) SDH activity (10 mM)	L. Frolich et al., 2004 [97]
Male wistar rat adult	Induced diabetic VaD	RSVL	20 mg/kg i.p. daily for 4 weeks	Cognition, body weight, blood glucose, carbachol, antioxidant levels, BDNF, and eNOS	Inflammation, HO-1, and NOX	Semil Selcen Gocmez et al., 2019 [98]
Male wistar rat adult	BCCAO	ALA	50 mg/kg i.p. daily for 28 days	Cognition, GSH levels, ACh, and ChAT	ROS and AChE levels	Ran-ran Zhao et al., 2015 [99]
Male SD rat 2 mo	BCCAO	RSVL	20 and 10 mL/kg i.p. daily for 4 weeks	Cognition and SOD levels	MDA and apoptosis	Yeqing Zhang et al., 2019 [100]
Male SD rat	Induced diabetic VaD	Beta-carotene	50 mg/kg oral daily for 15 days	Cognition and GSH levels	AChE and thiobarbituric acid reactive substance levels	Khian Giap Lim et al., 2022 [101]
Male SD rat	Induced diabetic VaD	Beta-carotene	100 mg/kg oral daily for 15 days	Cognition and GSH levels	AChE and Thiobarbituric acid reactive substances levels	Khian Giap Lim et al., 2022 [101]
Female SD rat 2 mo	BCCAO	Lycopene	50 mg/kg i.g. every other day for 2 mo	ROS in CA1, CA3, and DG	−−−	Ning-Wei Zhu et al., 2020 [102]
Female SD rat 2 mo	BCCAO	Lycopene	100 mg/kg i.g. every other day for 2 mo	Cognition and SOD activity	ROS in CA1, CA3, and DG	Ning-Wei Zhu et al., 2020 [102]
Female SD rat 2 mo	BCCAO	Lycopene	200 mg/kg i.g. every other day for 2 mo	ROS in CA1, CA3, and DG	−−−	Ning-Wei Zhu et al., 2020 [102]
Gerbil	BCCAO	Lycopene	20 mg/kg twice a day for 28 days	Cognition, neurons in CA1, and antioxidant activity	Inflammation, apoptosis, and GFAP in CA1	Wei Chen et al., 2021 [103]
Male SD rat 7–10 weeks	BCCAO	Lutein	0.5 mg/kg daily for 30 days	Cognition, pyramidal neuronal cells, and conduction in CA1	MDA levels	Hamideh Asadi Nejad et al., 2024 [104]
Male SD rat 7–10 weeks	BCCAO	Lutein	5 mg/kg	Cognition (NOR), pyramidal neuronal cells, and plasticity in CA1	MDA levels	Hamideh Asadi Nejad et al., 2024 [104]
Male mice	UCCAO	Astaxanthin	50 mg/kg daily for 30 days	Cognition (NOR), SOD levels, and IL-4 levels	IL1β levels	Ningwei Zhu et al., 2020 [105]
Male mice	UCCAO	Astaxanthin	100 mg/kg daily for 30 days	Cognition (NOR), IL4, and SOD levels	IL1β and MDA levels	Ningwei Zhu et al., 2020 [105]
Male mice	UCCAO	Astaxanthin	200 mg/kg daily for 30 days	Cognition (NOR and MWM), IL4, and SOD levels	IL1β and MDA levels	Ningwei Zhu et al., 2020 [105]
Male SD rat adult	BCCAO	Idebenone	100 mg/kg oral daily for 3 weeks	Cognition and neuronal levels in CA1	TNFα	Xudong Qian et al., 2021 [106]
Wistar rat 12–14 mo	BCCAO	RSVL	25 mg/kg oral for 4 weeks	Cognition, neurons, and antioxidant activity	Inflammation and MDA levels	Xingrong Ma et al., 2013 [107]
Male SD rat	BCCAO	RSVL	10 mg/kg oral daily for 4 weeks	Cognition, redox ratio, and SOD, HO1, and Nrf2 levels	ROS, GSSG, Hif1α, LPO, and protein carbonylation	Aarti Yadav et al., 2018 [108]
Male wistar rat	BCCAS	RSVL	40 mg/kg i.p. for 4 weeks	Cognition, synaptic spines, and synapse-associated proteins	−−−	Huagang Li et al., 2016 [109]
Female wistar rat 8–10 mo	mBCCAO	RSVL	Daily 10 mg/kg oral for 15 days	GSH and pyramidal neurons	MDA and GFAP levels	Veysel Haktan Ozacmak et al., 2016 [110]
Male SD rat	BCCAO	RSVL	50 mg/kg i.g. daily for 9 weeks	Cognition and antioxidant activity	AKT/mTOR signaling pathway, autophagy, apoptosis, and MDA levels	Nan Wang et al., 2019 [111]
Male wistar rat 3 mo	m2VO	RSVL	20 mg/kg i.p. daily for 7 days	Cognition, neuronal levels, and NGF	−−−	Janine R Anastacio et al., 2014 [112]
Male wistar rat	BCCAO	RSVL	10 mg/kg for 4 weeks	−−−	MDA, TNFα, and nitrite levels	Dongfang Shen et al., 2017 [113]
Male wistar rat	BCCAO	RSVL	20 mg/kg for 4 weeks	Cognition and GSH	MDA, IL1β,TNFα, AChE activity, and nitrite levels	Dongfang Shenet al., 2017 [113]
Male SD rat	BCCAO	Selenium	0.1 mg/kg p.o. daily for 4 mo	Cognition, posterior cerebral blood flow, neurons, synaptic plasticity, and GSH, NO, and SOD levels	ROS, NOX, and MDA level	Mo-li Zhu et al., 2023 [114]
Male ICR mice	UCCAO	Curcumin	20 µmol/kg i.g. daily for 2 weeks	Cognition (MWM) and GSH	ROS, SOD, and MDA levels	Runfang Zhang et al., 2021 [115]
Male ICR mice	UCCAO	Cur20	20 µmol/kg i.g. daily for 2 weeks	Cognition (MWM), GSH, and Hif1α	ROS, SOD, and MDA levels	Runfang Zhang et al., 2021 [115]
Male SD rats	Induced diabetic VaD	Curcumin	50 mg/kg i.p. every other day for 8 weeks	Cognition (MWM), NeuN, IL10, and IL4	Inflammation and apoptosis	Yaling Zheng et al., 2021 [116]

Abbreviations: SD, Sprague Dawley. MDA, malondialdehyde. GFAP, glial fibral acidic protein. SOD, super oxide dismutase. GSH, glutathione. i.p., intra-peritoneal. i.g., intragastric. UCCAO, unilateral common carotid artery occlusion. BCCAO, bilateral common carotid artery occlusion. m2VO, modified two-ventral occlusion. NGF, neuronal growth factor.

**Table 3:** Summary of clinical trials with antioxidants in vascular dementia.

Antioxidant	Treatment	Study details	Inference	Reference
Idebenone	45 mg/day b.i.d., for 120 days of oral administration	Randomized double-blind, placebo-controlled, and multicenter study on 108 elderly patients with mild-to-moderate mental deterioration of vascular origin	Improved acquisition and retention of verbal stimuli; improvements in memory, attention, and cognitive function	Vicenzo Marigliano et al., 1992 [127]
Idebenone and butyphthalide	Butyphthalide (0.2 g/time; 3 times/day) combined with idebenone (30 mg/time; 3 times/day) for 12 weeks	Retrospective clinical study of 126 patients with vascular dementia, average age of 67.3 ± 7.0 years	Improved daily living activities and cognitive function of patients	Hongxia Zhang et al., 2024 [169]
Idebenone and butyphthalide	−−−	Randomized observational study on 88 VaD patients	Combination of Butyphthalide with idebenone reduced serum inflammatory mediators levels in VaD patients, improved vascular endothelial functions and cognitive function	Fan Xing Qi et al., 2020 [170]
Huperzine A	Huperzine A (0.1 mg b.i.d.), 12 consecutive weeks	Randomized, double-blinded, and placebo-controlled study with 78 patients with mild-to-moderate VaD	Improved the cognitive function in patients with VaD	Zhi Qiang Xu et al., 2012 [171]
Idebenone	45 mg b.i.d. orally (90 mg over 6 mo)	Open multicenter study	Improved cognitive function in patients with VaD (MID and CCVD)	Giuseppe Nappi et al., 1992 [172]

Abbreviations: b.i.d., twice per day. CCVD, chronic cerebrovascular disorder. MID, multi-infarct dementia.

## Abbreviations

MHPG	3-methoxy-4-hydroxyphenylglycol
FeTPPs	4-sulfonatophenyl porphyrinato iron chloride
ALA	Alpha-lipoic acid
AD	Alzheimer’s disease
Aβ	Amyloid beta
BCCAO	Bilateral common carotid artery occlusion
BBB	Blood–brain barrier
BDNF	Brain-derived neurotrophic factor
CSF	Cerebral spinal fluid
CADASIL	Cerebral autosomal dominant arteriopathy with subcortical infarcts and leukoencephalopathy
CARASIL	Cerebral autosomal recessive arteriopathy with subcortical infarcts and leukoencephalopathy
CSF	Cerebral spinal fluid
CoQ10	Coenzyme Q10
eNOS	Endothelial nitric oxide synthase
ERK	Extracellular signal-regulated kinase
GSH	Glutathione
CA1	Hippocampal circuit 1
HVA	Homovanillic acid
H_2_O_2_	Hydrogen peroxide
HIF-1α	Hypoxia inducible factor-1
ICAM	Intercellular adhesion molecule 1
LDL	Low-density lipoprotein
MDA	Malondialdehyde
MMP	Matrix metalloproteinase
MitoQ	Mitoquinone mesylate
MWM	Morris water maze
NADPH	Nicotinamide adenine dinucleotide phosphate
NO	Nitric oxide
NFkB/NF-κB	Nuclear factor kappa light chain enhancer of activated B cells
Ox-LDL	Oxidized LDL
ONOO−	Peroxynitrite
ROS	Reactive oxygen species
RSVL	Reservatrol
5-HIAA	Serotonin
SNP	Single-nucelotide polymorphism
SDH	Succinate dehydrogenase
O_2_−	Superoxide
SOD	Superoxide dismutase
BH4	Tetrahydrobiopterin
UCCAO	Unilateral common carotid artery occlusion
VCAM-1	Vascular cell adhesion molecules
VaD	Vascular dementia
RSVL	Resveratrol
